# Research on Nondestructive Testing Method Based on Magnetic Characteristics of Electron Beam Weld Defects

**DOI:** 10.3390/s25134094

**Published:** 2025-06-30

**Authors:** Qiangqiang Cheng, Jijun Liu, Yisong Wang, Guisuo Xia, Chunquan Li

**Affiliations:** 1 School of Mechanical and Electronic Engineering, Gandong University, Fuzhou 344000, China; 2 The Key Laboratory of Nondestructive Testing, Nanchang Hangkong University, Nanchang 330031, China; lukasong77@163.com (Y.W.); xiaguisuo@163.com (G.X.); 3 School of Information Engineering, Nanchang University, Nanchang 330031, China; lichunquan@ncu.edu.cn

**Keywords:** aluminum alloy, electron beam welding, weak magnetic detection, wavelet transform, finite element analysis

## Abstract

In view of the problems of poor safety, slow detection speed, and low accuracy of existing nondestructive testing (NDT) technologies, such as X-ray methods and ultrasonic detection in detecting electron beam weld defects in aluminum alloys, this study proposes a weak magnetic NDT method based on the geomagnetic field. Firstly, the finite element analysis method was used to establish a simulation model of aluminum alloy electron beam welding defects, and the distribution characteristics of the magnetic field around weld defects, such as cracks and pores, were obtained. Then, the magnetic anomaly signal at the crack weld was identified by combining the wavelet transform and the least squares method. Finally, experimental tests show that the proposed method can safely, quickly, and accurately detect the defects of aluminum alloy electron beam welds.

## 1. Introduction

Aluminum alloy, one of the most widely used light metal materials, exhibits excellent electrical conductivity, thermal conductivity, and corrosion resistance under specific process conditions. Additionally, it offers advantages such as low density, high strength, and good plasticity, making it widely applicable in industries such as aerospace, automotive, machinery manufacturing, shipping, and petrochemicals [1,2,3]. Electron beam welding is a commonly employed method for welding aluminum alloy structural parts, particularly in vehicle bodies [4].

Electron beam welding is one of the most advanced processing methods among high-energy beam welding techniques. This type of fusion welding utilizes a high-speed electron stream that converges in a high-vacuum environment to bombard the weld of a workpiece, causing the material to rapidly heat and melt. The material then undergoes rapid cooling to form a continuous weld at the joint [5]. Compared with traditional welding methods, electron beam welding offers several advantages: concentrated energy, fast welding speed, a large depth-to-width ratio of the welded joint, a small heat-affected zone, minimal welding deformation, high mechanical properties of the joint, controlled input weld energy, and reliable protection. However, during the welding process, improper selection of welding parameters, excessive or insufficient heat input, and other factors can lead to defects such as pores, slag, cracks, lack of fusion, and incomplete penetration in the welded joint [6,7]. The presence of these weld defects directly affects the performance of structural components. Therefore, nondestructive testing of electron beam weld seams is typically required to ensure weld quality [8].

Much research on electron beam weld defect detection technology has been performed both domestically and internationally, and the commonly used nondestructive testing methods are ultrasonic testing and X-ray testing [9,10,11]. X-ray testing, especially real-time imaging and industrial computed tomography (CT), is intuitive, and it is easy to record the display of the defects. In [12], the authors presented three-dimensional imaging and a quantitative analysis of pores in electron beam-welded joints of dissimilar steels with Fe-Al alloys using X-ray tomography. In [13], TOFD acoustic beam coverage was calculated, and a TOFD inspection of electron beam welds based on a self-developed system was performed. The experiments showed that the ultrasonic time-of-flight diffraction (TOFD) inspection method can detect electron beam welds quickly and accurately. In [14], bonding and electron beam welding quality control of aluminum stabilized and reinforced compact muon solenoid (CMS) conductors using the ultrasonic phased array technique. These inspection methods, although very mature, have certain drawbacks. X-ray inspection has the advantages of high sensitivity, visual display of defects, and repeatability, and the results can be stored for a long time [15]. However, it is not sensitive to longitudinal cracks in the weld, which may lead to leakage, and its penetration into thick plates is limited. Ultrasonic inspection methods include water-immersion ultrasonic C-scan, air-coupled ultrasonic inspection, and phased-array ultrasonic inspection. Although these methods can detect electron beam welds, their detection speed is slow and their efficiency is low, which cannot meet the requirements for the rapid inspection of large electron beam welds. Additionally, the reliability of these inspection methods is not high, ranging from approximately 50% to 70% [16]. In [17], magnetic methods are investigated as an indirect means of assessing carburization depth to avoid failure and severe degradation of high-performance mechanical parts. In [18], a novel Dynamic Permeability Testing (DPT) method is proposed for detecting internal defects in ferromagnetic materials. The above studies illustrate that nondestructive testing methods based on magnetic theories have significant practical application value.

This paper presents a weak magnetic nondestructive inspection technique for detecting electron beam weld defects in aluminum alloys based on the geomagnetic field [19]. This technique is simple to operate, does not require a coupling agent or excitation equipment, is less affected by the lift-off effect, and offers fast detection speed and high accuracy. Firstly, finite element analysis was used to establish a simulation model of weak magnetic field weld defects, including cracks and porosity, in aluminum alloy electron beam welding specimens. The relationship between defect size and magnetic anomaly characteristics was then analyzed. Second, a weak magnetic detection test of cracked aluminum alloy electron beam welds was conducted under laboratory conditions, and the magnetic anomaly signals were collected. Finally, the multiscale wavelet transform was employed to separate the defect signal features from the acquired raw magnetic induction intensity signal, allowing for more intuitive identification of the weld defects.

## 2. Principle and Method of Weak Magnetic Detection

Magnetism is prevalent in the space we live in. In fact, the Earth itself is a large magnet, and microscopic particles also exhibit magnetic properties, making magnetic phenomena very common. When a material is placed in a magnetic field, it exhibits magnetism; this process, in which a nonmagnetic material acquires magnetism, is called magnetization. The magnitude of a material’s magnetism under the influence of an external magnetic field is referred to as magnetization strength. For a given material, the greater the strength of the external magnetic field, the greater the degree of magnetization. The formula is expressed as follows:

(1) M=χH

where M is the magnetization strength (A/m); H is the external magnetic field strength (A/m); and χ is the magnetization rate of the material, which can be positive or negative, and is related to the nature of the material itself.

When the magnetization of the magnetic medium in the magnetic field occurs, an additional magnetic field is generated, and the relationship between the total magnetic induction strength after the superposition of the additional magnetic field and the original magnetic field and the original magnetic field strength can be expressed as follows:

(2) $$B=\mu_{0}H+\mu_{0}M=(1+\chi )\mu_{0}H=\mu_{r}\mu_{0}H$$

where B is the magnetic induction strength (T); $\mu_{0}$ is the vacuum permeability, the size of which is $4\pi \times 10^{-7}$ H/m; and $\mu_{r}$ is the relative permeability, a physical quantity used to characterize the degree of magnetization of a substance in an external magnetic field, which is related to the magnetization rate as $\mu_{r}=1+\chi$.

With the same external magnetic field strength, the magnetization strength and magnetic induction strength are influenced only by the magnetization rate. According to the magnetization rate of materials, they can be classified into five major categories: ferromagnetic, paramagnetic, antimagnetic, subferromagnetic, and antiferromagnetic. The geomagnetic field is equivalent to the natural magnetization field, and the materials in the natural environment show certain magnetic properties under the magnetization of the geomagnetic field; therefore, any material is a magnetic medium, and its magnetization depends on the relative magnetic permeability $\mu_{r}$ of the material itself. If there are two different magnetic media in the geomagnetic field, due to the different relative permeabilities of these two magnetic media, the direction of the magnetic induction lines will change when the magnetic induction lines pass through their dividing interface [20], similar to the refraction of light, the angle of incidence $\theta_{1}$ of the magnetic induction lines in the first magnetic media with relative permeability $\mu_{r1}$, and the angle of refraction $\theta_{2}$ in the second magnetic media with relative permeability $\mu_{r2}$, which satisfy the following relationship:

(3) $$\frac{\tan\theta_{1}}{\tan\theta_{2}}=\frac{\mu_{r1}}{\mu_{r2}}$$

**Figure 1:**
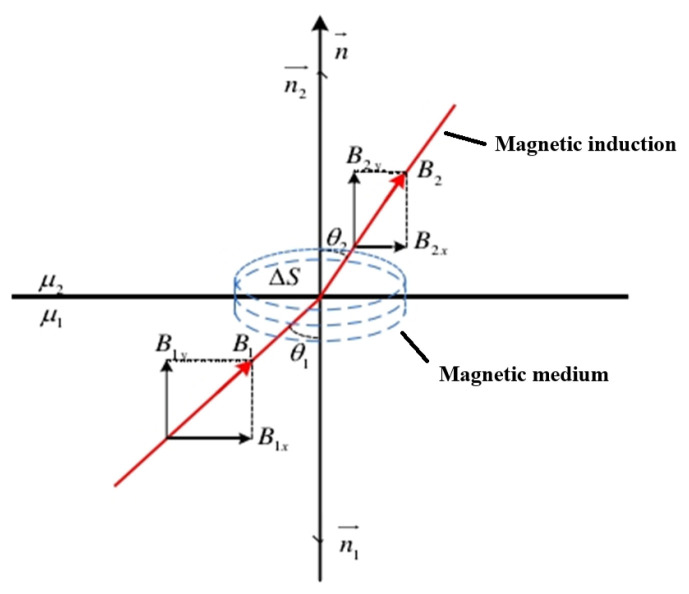
Physical model of the refraction of magnetic induction lines.

The physical model of the refraction of magnetic induction lines is shown in Figure 1.

**Figure 2:**
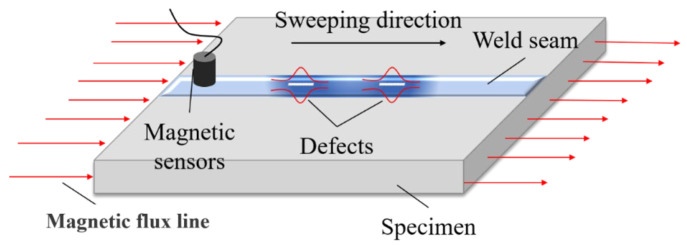
Principle of weak magnetic detection.

Aluminum alloys exhibit a certain weak paramagnetism due to magnetization by the geomagnetic field, which slightly enhances their own magnetic induction strength [21]. When the internal organization of the aluminum alloy electron beam weld located in the geomagnetic field is uniform and continuous, its magnetic permeability is the same, the magnetic induction lines are uniformly distributed in the material, and the magnetic induction intensity signal on the surface of the weld is measured by using a high-precision magnetic sensor changes more gently without obvious drastic fluctuations. If there are discontinuous defects (such as porosity and cracks) inside the weld, the magnetic permeability at the defect will change; therefore, it will macroscopically manifest as the distortion of the magnetic induction intensity signal on the surface of the weld, which is called a magnetic anomaly [22,23]. The defect identification of aluminum alloy electron beam welds can be achieved through the detection of abnormal magnetic induction intensity signals using high-precision magnetic sensors. A schematic diagram of the weak magnetic detection principle is shown in Figure 2.

The magnetic anomaly signal can be measured using the magnetic field measurement sensor, and the weak field detection flaw detector used in this paper is mainly composed of four parts: signal acquisition control module, magnetic field measurement sensor, host computer, and data processing software. When the magnetometer sensor picks up a weak magnetic signal from the surface of the test block, the data acquisition module converts the signal into an analog signal, which is then transferred to the integrated circuit board. The analog signal is then converted to a digital signal via an A/D converter. The digital signal is transmitted to the weak magnetic detection host computer at a transmission rate of 10 Mbps via the Ethernet UDP transmission protocol, and then the data are displayed and postprocessed in real time using the data processing software within the computer. The weak magnetic probe uses a high-precision single-component fluxgate sensor with a range of ±250,000 nT, and the resolution can be accurate to 0.1 nT with a sampling frequency of 12.5 Hz.

In the magnetic sensor probe close to the weld surface sweeping process, due to the impact of the weld surface roughness, the probe will inevitably experience a slight vibration; in addition, the superposition of the external magnetic field will produce some interference in the magnetic induction intensity curve of the defect signal extraction. Therefore, in the weak magnetic detection of aluminum alloy electron beam welds, the collected magnetic induction signal is actually a complex signal formed by the superposition of multiple signals, including not only the magnetic field distribution signal of the weld itself and the defect anomaly signal, but also the vibration signal during the detection process and the background noise interference signal. To reduce the interference and effectively extract the signal characteristics of defects in aluminum alloy electron beam welds, the wavelet transform is used to extract the defect signal. The wavelet transform is suitable for detecting transient anomalies entrained in normal signals and has unique advantages for processing nonsmooth signals [24].

Composite signals have been shown to be able to be decomposed into multiple subsignals at different scales using wavelet basis functions [25]. The aluminum electron beam weld defect signal can be understood as an abrupt signal present in the geomagnetic field signal, and the wavelet transform is very suitable for the local abruptness of the signal. The most critical issue is the choice of wavelet basis; the selection of different wavelet bases for the analysis and processing of the signal has different effects, and a suitable wavelet basis is more beneficial for the identification of the characteristic signal [26]. The Daubechies wavelet is a wavelet function that can be used for both continuous and discrete wavelet transforms and has been widely used in NDT. Therefore, after analysis and comparison, the db6 wavelet with a higher N value is selected to perform 3-scale decomposition of the detection signal in this paper.

To show the relationship between the magnetic induction intensity signal and the crack depth more intuitively, quadratic curve fitting was performed by the least squares method, which is simple to calculate and has good wholeness and stability. The crack amplitude ∆B and depth h were fitted using least squares fitting with the following equation:

(4) $$\Delta B=2.1875h^{2}-1.325h+4.9$$

## 3. Simulation Analysis

### 3.1. Finite Element Geometry Modeling

**Figure 3:**
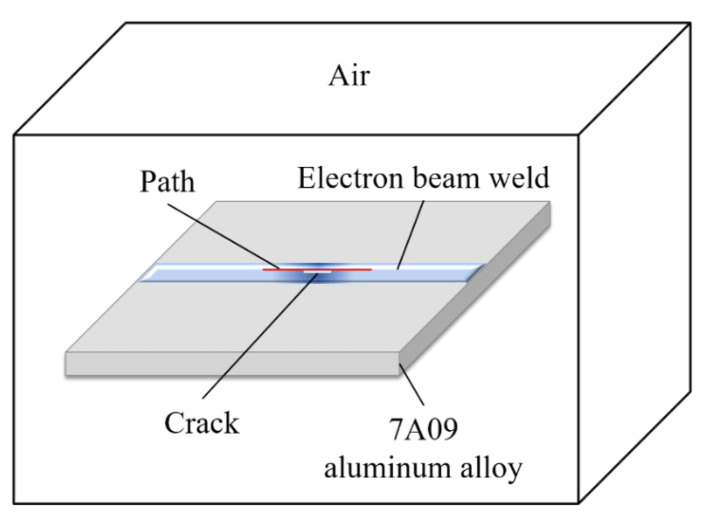
Finite element simulation solid model.

The solid modeling method is used to establish the electron beam weld weak magnetic detection finite element analysis model, whose structure is composed mainly of several parts, such as an aluminum alloy plate with an electron beam weld, weld defects, air layer, etc. [27]. The finite element geometry model is shown in Figure 3. The thickness of the 7A09 aluminum alloy in the model is 5 mm, the length is 110 mm, the width is 80 mm, the air domain size is 200 mm × 160 mm × 140 mm, and the weld width is 2 mm. The crack is simplified to a rectangular groove defect in the center of the surface of the electron beam weld of 7A09 aluminum alloy.

After establishing the solid model and setting the cell type, the material properties of the weldment are set according to the physical conditions, materials, and parameters of the test. The key components in the model are the aluminum alloy plate, weld seam, defect, and air domain, and the material properties of these components need to be defined separately in the model. In this paper, 7A09 aluminum alloy is used as the research object, and the defect is air. The relative permeability size of air is known to be 1.0, and the relative permeability of 7A09 aluminum alloy is 1.00002.

**Figure 4:**
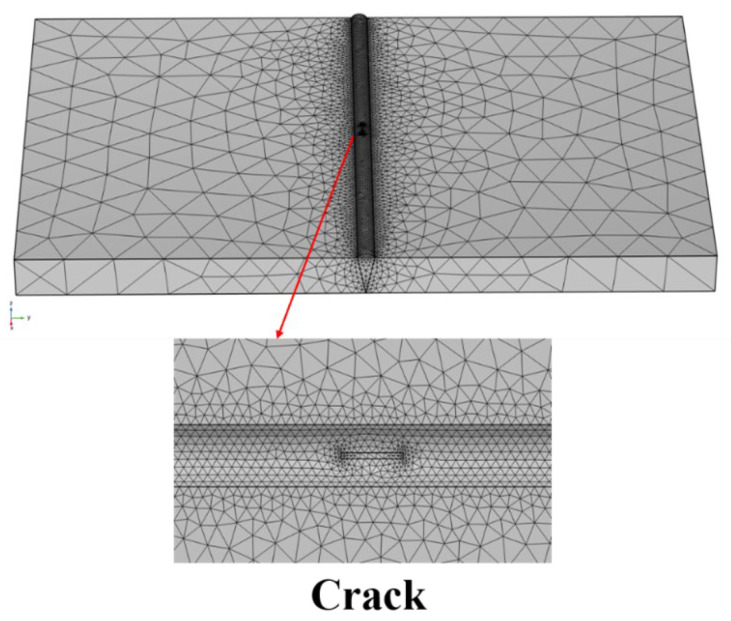
Finite element 3D model meshing.

In areas where the magnetic field does not vary drastically, a larger grid spacing can shorten the computer running time; for more accurate solution results and areas where the magnetic field varies drastically, the grid density needs to be slightly tighter, but the required computational time and computational memory are greatly increased. The rationality of grid division is even more important when calculating using 3D models; otherwise, the rationality of grid division will lead to nonconvergence of the model calculation results. In this paper, we use the physical field control grid method of meshing: the cell size is hyperfine, the electron beam welding defects of the finite element 3D model of the mesh division are shown in Figure 4, and the weld uses a uniform dense mesh to improve the accuracy of the calculation. Three mesh densities (coarse mesh of 0.5 mm, medium mesh of 0.2 mm, and ultra-fine mesh of 0.1 mm) were used to calculate the amplitude ∆*B* when the crack depth was 1 mm. The results were 32.1 nT, 33.8 nT, and 34.1 nT, respectively, and the relative error was less than 3% (Table 1). This indicates that the ultra-fine mesh (0.1 mm) has reached the saturation of calculation accuracy, and further mesh refinement has a negligible impact on the results (in line with the mesh independence principle of finite element analysis). The number of elements and element sizes in the region are shown in Table 2. This meshing strategy balances computational accuracy and resource consumption, in line with the best practices of finite element analysis.

### 3.2. Parameter Settings of the Geomagnetic Field

Geomagnetic anomalies represent the deviation between the natural static magnetic field and the predicted value of the Earth’s magnetic field based on the magnetic dipole model. Static magnetic problems with negligible currents can be solved using magnetic scalar potentials. Variations in magnetic permeability cause small deviations from standard values (local geomagnetic anomalies), which can be accurately modeled using the approximate magnetic potential formulation of the “magnetic field, no current” interface. In the simulation domain, the background magnetic field is assumed to be uniform.

In the no-current region, the equation is as follows:

(5) ∇×H=0

The magnetic scalar potential $V_{m}$ can be defined and is obtained as follows:

(6) $$H=-\nabla V_{m}$$

This calculation is similar to the definition of the potential of an electrostatic field. Using the intrinsic relationship between the magnetic induction strength and the magnetic field, the following was obtained:

(7) $$B=\mu H+B_{r}$$

(8) ∇⋅B=0

The equation for $V_{m}$ can be derived as follows:

(9) $$\nabla \cdot (-\mu \nabla V_{m}+B_{r})=0$$

The approximate potential formula used in this model splits the total magnetic potential into external and approximate potentials: $V_{tot}=V_{ext}+V_{red}$, where the approximate potential $V_{red}$ is the dependent variable:

(10) $$\nabla \cdot (-\mu \nabla (V_{ext}+V_{red})+B_{r})=0$$

The external magnetic potential is more easily defined as the external magnetic field; therefore, the actual equation used is as follows:

(11) $$\nabla \cdot (-\mu \nabla (V_{red})+B_{r}+\mu H_{ext})=0$$

To simulate the background magnetic field, the external magnetic field components are represented by the total intensity, magnetic declination, and magnetic inclination as follows:

(12) extHOx=HO⋅cos(Incl)⋅sin(Decl)

(13) extHOy=HO⋅cos(Incl)⋅cos(Decl)

(14) extHOz=−HO⋅sin(Decl)

The magnetic inclination and magnetic declination angles are approximately Incl = 59.357° and Decl = 12.275°, respectively, and the magnitude of the natural magnetic induction $\left(B_{0}=\mu_{0}H_{0}\right)$ is estimated to be 48.163 μT.

### 3.3. Analysis and Discussion of Simulation Results

**Figure 5:**
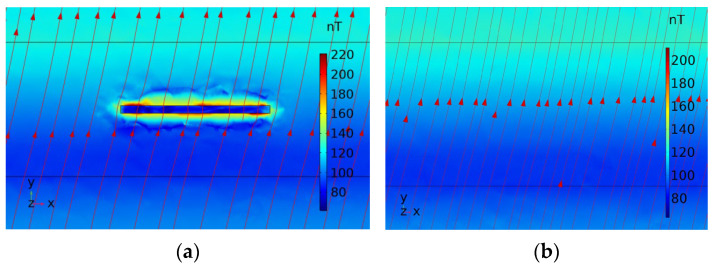
Cross-sectional clouds of magnetic induction intensity with and without defects: (**a**) defective; (**b**) no defects.

The distribution of magnetic induction lines and magnetic induction intensity clouds for the aluminum alloy plate cross-section, both with and without defects, is shown in Figure 5. The figure indicates that the distribution of magnetic induction intensity at the defect-free weld seam is uniform and remains essentially unchanged. The magnetic induction lines pass uniformly through the weld seam, forming a stable magnetic field, with the lines parallel to each other. In the presence of defects, the magnetic induction lines become significantly distorted, and the magnetic induction intensity cloud is unevenly distributed at the defect sites. This demonstrates that the weak magnetic detection technique is theoretically feasible and effective.

**Figure 6:**
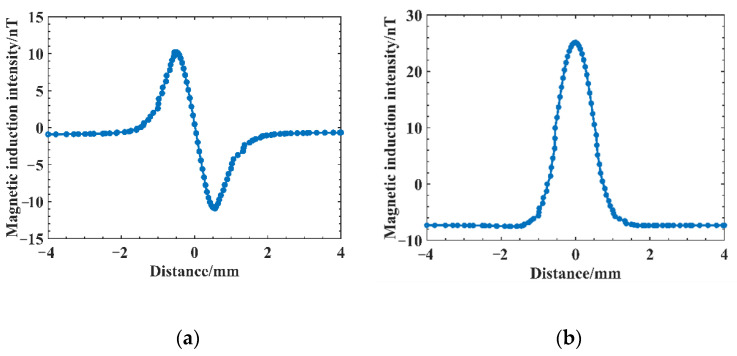
Magnetic induction intensity distribution curve at the defect: (**a**) magnetic induction $B_{x}$; (**b**) magnetic induction $B_{z}.$

To determine the distribution of magnetic induction intensity at the weld defect, we set the defect length as 1 mm, width as 0.2 mm, depth as 1 mm, and height along the weld surface as 0.1 mm for path extraction and derived the distribution curves of magnetic induction intensity $B_{x},B_{z}$ at the points 4 mm from each side of the defect center point, as shown in Figure 6. From the curve, the radial component of the magnetic induction $B_{x}$ distribution curve at the defect shows two peaks centered at the origin, first positive and then negative, and symmetrically distributed on both sides of the defect center, with a maximum at the edge of the defect length. $B_{z}$ shows a clear convex peak at the defect with positive polarity, and its value increases, i.e., the magnetic induction intensity increases at the defect, because when there is a discontinuous defect inside the weld, the defect is filled with air, and these defects will cause a change in magnetic permeability. The magnetic permeability of the air at the defect is less than the permeability of the weld itself. The magnetic field hindrance at the defect site will become larger, the magnetic induction lines at the defect will be repelled to bend, the magnetic induction line density at the defect will decrease, and the magnetic induction line density above the defect will become larger, which leads to the defect. The magnetic induction line density at the weld surface above increases; therefore, the magnetic induction intensity $B_{z}$ curve appears as a convex magnetic anomaly, and the simulation results are consistent with the theory of weak magnetic detection.

**Figure 7:**
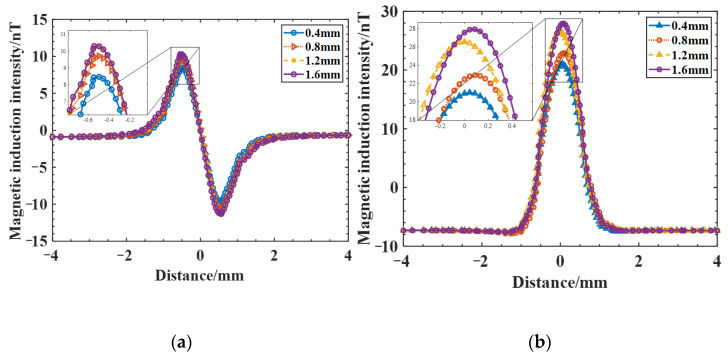
Magnetic induction intensity distribution curves for cracks of different depths: (**a**) magnetic induction $B_{x}$; (**b**) magnetic induction $B_{z}.$

**Figure 8:**
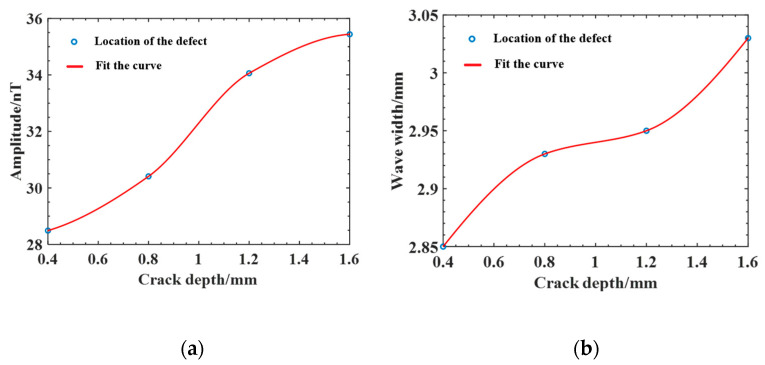
Variation curve of the magnetic anomaly characteristic amount of cracks with different depths: (**a**) Amplitude; (**b**) Wave width.

In actual production, the width of the electron beam weld is generally small, the crack width is extremely small, and larger width cracks do not easily appear; therefore, the preset crack widths are taken as 0.2 mm to simulate real fine crack defects, while the length and depth direction crack expansion is larger. To compare and analyze the effect of crack depth and length on the magnetic field distribution, cracks with depths of 0.4 mm, 0.8 mm, 1.2 mm, and 1.6 mm were established at a crack length of 1 mm for comparative analysis, and the magnetic induction intensity $B_{x}$ and $B_{z}$ distribution curves for cracks of different depths were obtained. From the results in Figure 7, the magnetic induction intensity $B_{x}$ also shows two typical peaks, one positive and one negative, and is symmetrically distributed on both sides of the crack center, and the magnetic induction intensity $B_{z}$ shows a typical convex peak. As the crack depth increases, the weak magnetic signal is enhanced, and the peaks of magnetic induction intensity $B_{x}$ and $B_{z}$ both increase rapidly at first, and then the increasing trend becomes slower; however, the peak increase in $B_{x}$ is smaller, the peak spacing of one positive and one negative peak of curve $B_{x}$ does not change significantly, and the wave width ∆L (the distance between the lowest points of the signal curve around the peak) of curve $B_{z}$ changes less. As summarized in Table 3, the wave width ∆L and amplitude ∆B (the difference between the peak value $B_{max}$ and the minimum value $B_{min}$ of the magnetic anomaly) of the magnetic induction intensity curve $B_{z}$ at different depths of cracks are shown in Figure 8. The amplitude ∆B first increases rapidly with increasing crack depth, and then the increasing trend becomes slower, and the wave width ∆L also increases, although the overall change is small.

**Figure 9:**
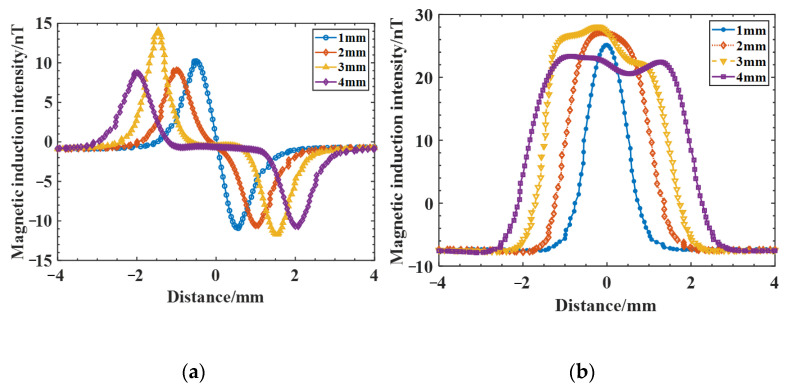
Magnetic induction intensity distribution curves for cracks of different lengths: (**a**) magnetic induction $B_{x}$; (**b**) magnetic induction $B_{z}.$

Cracks with lengths of 1 mm, 2 mm, 3 mm, and 4 mm were established at a crack depth of 1 mm for comparative analysis, and the magnetic induction intensity $B_{x}$ and $B_{z}$ distribution curves for different lengths of cracks were obtained (Figure 9). From the results in Figure 9, the peak value of magnetic induction $B_{x}$ varies less at the same depth with different lengths; however, as the crack length increases, the peak spacing of the two peaks of the curve $B_{x}$ positively and negatively increases, the wave width ∆L of magnetic induction $B_{z}$ also gradually becomes larger, and the peak value of $B_{z}$ increases first and then decreases with the increasing crack length.

**Figure 10:**
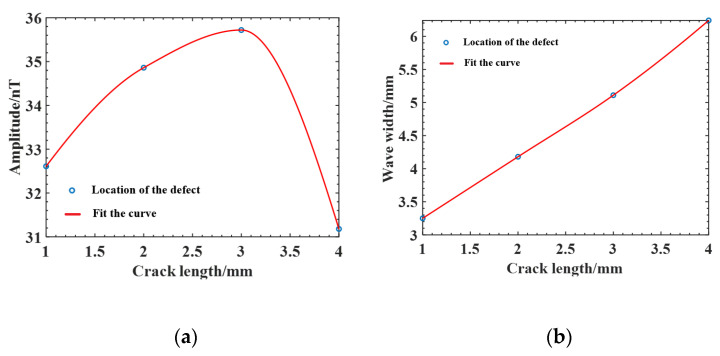
Variation curve of the magnetic anomaly characteristic number of cracks with different lengths: (**a**) amplitude; (**b**) wave width.

As summarized in Table 4, the wave width ∆L and amplitude ∆B of the magnetic induction intensity curve $B_{z}$ for different lengths of cracks are shown in Figure 10. The amplitude ∆B increases and then decreases as the crack length increases, and the wave width ∆L increases approximately linearly.

**Figure 11:**
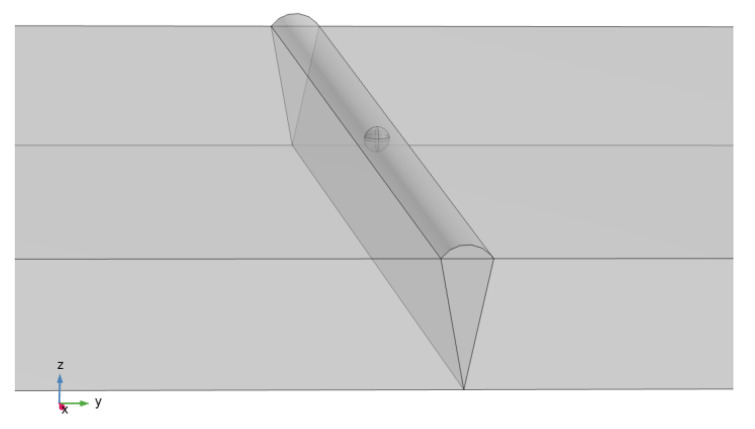
Pore defect model. The diagram is generated using COMSOL 5.6.

In order to verify the versatility of the method, a model of pore defects was established, which is actually shown in Figure 11. The pore model is simplified to spherical defects with diameters of 0.5 mm, 1.0 mm, 1.5 mm, and 2.0 mm. The relative permeability of pores is *μ_r_* = 1.0, and the weld material *μ_r_* = 1.00002 (consistent with the crack defect model).

Table 5 shows the magnetic anomalies of pores with different diameters, and it can be seen that the aperture is positively correlated with the amplitude of the magnetic induction intensity, which is consistent with the crack defect law, which verifies the versatility of the method.

## 4. Experimental Analysis

The test specimen used in this study is a 7A09 aluminum alloy rolled plate with prefabricated defects provided by China Hubei Sanjiang Aerospace Wanshan Special Vehicle Co., Ltd. The welding method uses a flat plate, but with welding, and the size of the weld is 110 mm × 80 mm × 5 mm. Its main chemical composition is shown in Table 6.

Using the laboratory’s self-developed weak magnetic detection flaw detector to test the above aluminum alloy specimen, a high-precision magnetic sensor is used to sweep the surface of the aluminum alloy electron beam weld, and if background noise is not considered, the signal collected is the normal component $B_{z}$ of the surface magnetic induction intensity of the weld after magnetization in the geomagnetic field.

**Figure 12:**
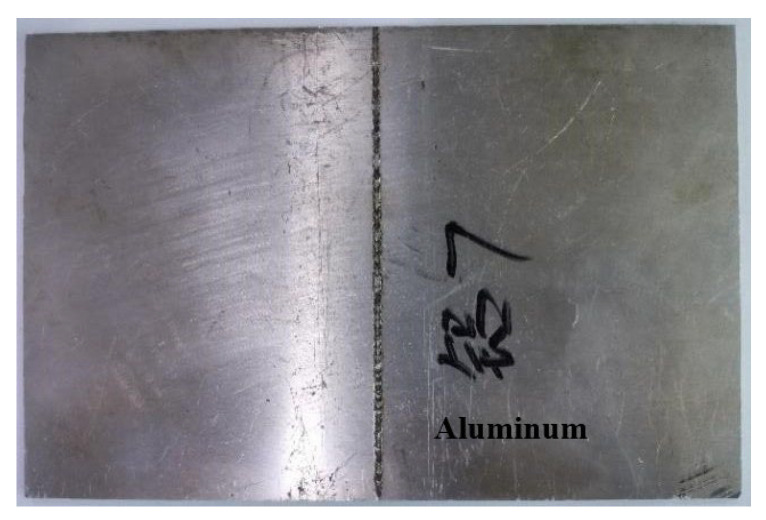
Aluminum alloy electron beam-welded specimen.

**Figure 13:**
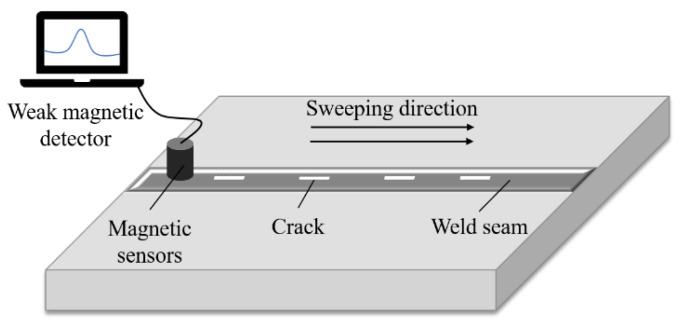
Schematic diagram of specimen testing.

During the testing process, the aluminum alloy specimen is first placed in a uniform and stable magnetic field environment to ensure that there is no strong magnetic interference within a 2 m radius around the specimen. The weak magnetic probe is placed close to the surface of the aluminum alloy electron beam weld and swept from left to right along the weld direction without lifting away from the ground at a uniform and stable speed, and the magnetic induction signal is continuously collected during the sweeping process. To avoid uncertainties in the detection process, the sweeping process is repeated at least twice. The aluminum alloy electron beam welded specimen used in the test is shown in Figure 12, and a schematic diagram of the test specimen is shown in Figure 13. A total of four linear defects were machined to simulate real cracks, with prefabricated defects of 1 mm in length, 0.2 mm in width, and 1.2 mm, 1.6 mm, 0.8 mm, and 0.4 mm in depth from left to right.

**Figure 14:**
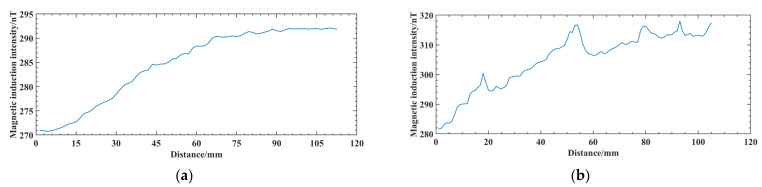
Detection signals of electron beam welded specimens of aluminum alloys: (**a**) aluminum alloy surface without defects; (**b**) electron beam weld surface of aluminum alloy.

After the inspection is completed, the magnetic induction signal collected by the weak magnetic sensor is shown in Figure 14. To compare the signal difference between the test block at the defect and defect-free areas, (a) is the magnetic induction signal collected by the sensor at the defect-free area of the aluminum alloy surface, and (b) is the magnetic induction signal collected at the surface of the aluminum alloy electron beam weld.

In Figure 14a curve, the magnetic induction intensity changes relatively smoothly, with no significant abnormal fluctuations. In Figure 14b, the curve can be seen at sweep distances of 15−22 mm, 47−58 mm, 76−86 mm, and 90−100 mm, which appear as convex magnetic anomalies, consistent with the simulation results. In addition, there are still some deviations between the apparently fluctuating raised positions on the curve and the actual defect positions at 20, 50, 80, and 100 mm on the weld, which are caused by the uneven speed of the manual sweep and the influence of the roughness of the weld surface during the magnetic sensor travel, showing that the use of weak magnetic detection technology is initially feasible for the detection of defects in aluminum alloy electron beam welds.

**Figure 15:**
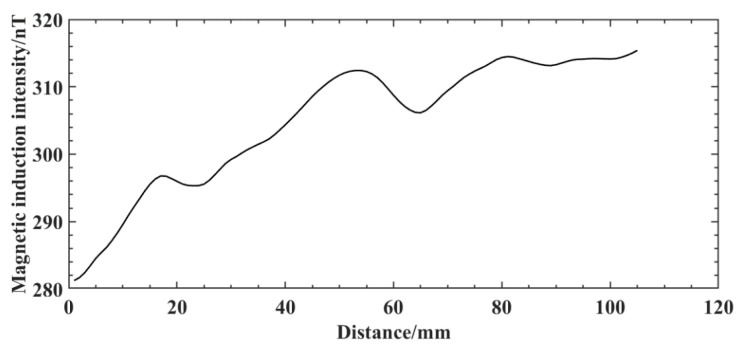
Low-frequency signal separated from the original signal of the weld.

**Figure 16:**
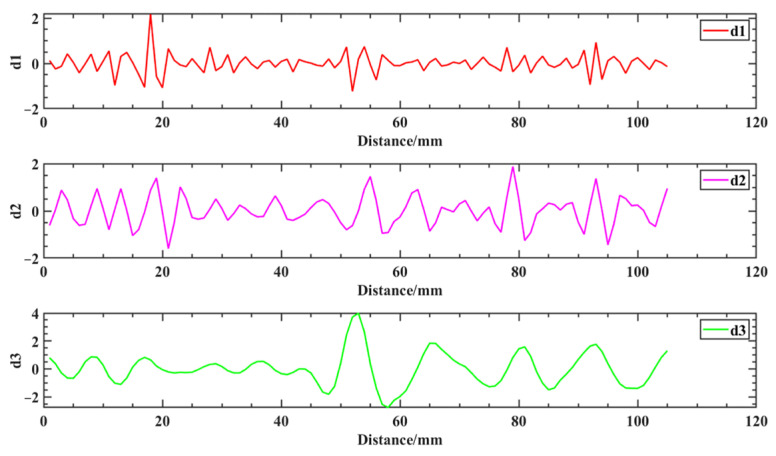
Multiscale wavelet decomposition signal.

The wavelet transform is used to extract the defective signal. First, the approximate coefficients of the original magnetic induction intensity signal of the weld are reconstructed in a single branch to obtain the low-frequency signal waveform graph shown in Figure 15. From the comparison between Figure 14b and Figure 15, the waveforms between the separated low-frequency signal and the original signal of the weld are slightly different, but the overall change trend is the same. By reconstructing the detail coefficients separately, the high-frequency components of the 3-scale decomposition of the original magnetic induction intensity signal of the weld are obtained, as shown in Figure 16. From the analysis of the decomposition results, d2 exhibits a staged high-frequency variation characteristic, which is due to the high-frequency noise interference caused by the slight jitter generated during the probe sweeping process due to the influence of the surface roughness of the aluminum alloy electron beam weld. Additionally, due to the noise interference of the background environment in which it is located, d3 shows a weak near-sinusoidal periodic variation. The weld defect signal exists mainly on d1, and there is a clear degree of distinction between the defect signal and the interference signal, in which significant abnormal signal fluctuations occur at sweep distances of 17−21 mm, 50−56 mm, 77−82 mm, and 91−98 mm, respectively. The changes are extremely sharp compared to the signals in the neighboring areas, and the four artificial defects on the aluminum alloy electron beam welding specimens are clearly presented.

Therefore, the method of multiscale wavelet transform processing, after choosing a suitable wavelet basis, can accurately and effectively extract the weld defect signal from the complex original magnetic signal.

**Figure 17:**
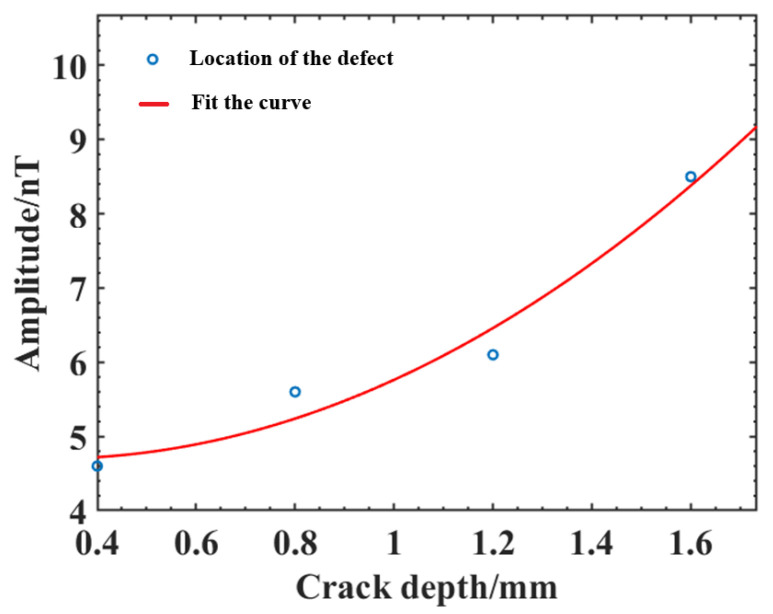
Least squares fitted depth curve.

Table 7 shows the amplitude ∆B and the crack depth h for each crack location. The curve fitting coefficient is 0.965, and the correction fitting coefficient is 0.8949, which is a good fit with high accuracy and within the allowable error. The least squares fitted depth curve is shown in Figure 17, which shows that the crack depth h is approximately positively correlated with the crack amplitude ∆B within a certain range, and the deeper the crack depth is, the larger the amplitude, which is consistent with the simulation analysis results. Therefore, the weak magnetic detection technique can achieve the identification and fixing depth of cracks in the electron beam welds of aluminum alloys.

**Figure 18:**
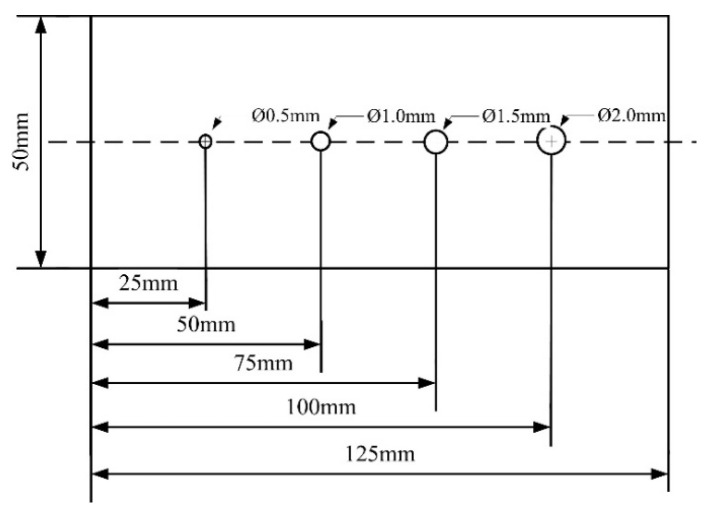
Pore defect setting.

**Figure 19:**
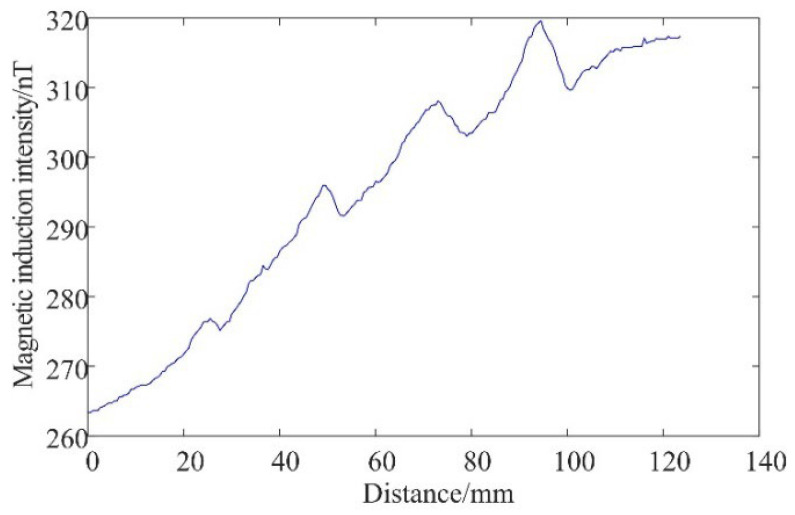
Pore defect detection signal results.

In order to further verify the versatility of the weak field detection, the pore defects shown in Figure 18 were set. Pore defects with diameters of about 0.5 mm, 1.0 mm, 1.5 mm, and 2.0 mm are set at 25 mm, 50 mm, 75 mm, and 100 mm on aluminum alloy plates, respectively. The results of the scan are shown in Figure 19. It can be seen that the position of the magnetic field anomaly corresponds to the position of the pore defect, and the increment of the magnetic field anomaly is close to the simulation results, which verifies the versatility of the method.

## 5. Conclusions

This paper adopts the finite element analysis method to establish a weak magnetic field weld defect simulation model for 7A09 aluminum alloy electron beam welding specimens, revealing the quantitative relationship between the crack/pore size and magnetic anomaly characteristics (ΔB, ΔL).

By combining the wavelet transform and the least squares method, the high-precision extraction of defect signals is achieved (fitting coefficient R^2^ = 0.965).

Experiments have verified the detection ability of this method for cracks above 0.4 mm and pores above 0.5 mm, with higher accuracy than traditional ultrasonic and X-ray methods.

In the current experiment, the minimum detectable depth is 0.4 mm. However, due to the limitation of the sensor resolution (0.1 nT), the defect signals shallower than 0.4 mm may be submerged by noise. It is necessary to further optimize the sensor accuracy or adopt the phase-locked amplification technique.

The surface roughness of the weld seam can cause fluctuations in the sensor lift-off effect, leading to an increase in the standard deviation of the B_z signal (in the experiment, the ΔB fluctuates by ±1 nT). Interference can be reduced by sandpaper polishing or adaptive filtering algorithms (such as Kalman filtering), and relevant optimization plans have been included in the future research plan.

## 6. Future Research

This paper uses rectangular grooves to simulate real cracks, mainly to control variables for a quantitative analysis of the influence of depth and length. Actual irregular cracks can be regarded as the superposition of rectangular grooves in multiple directions, and their magnetic anomaly signals can be detected through the signal fusion of multi-direction scans (such as 0°/45°/90°). Future research will introduce a fractal geometry model to simulate the irregular contours of real cracks, so as to improve the engineering applicability of the model.

Future work should focus on correlating magnetic anomaly characteristics (such as ΔB) with fracture mechanics parameters (such as stress intensity factor K) and validating this method under cyclic loading. Moreover, weak magnetic detection could be combined with acoustic emission or strain monitoring to achieve the real-time fatigue prediction of key welded structures.

## Figures and Tables

**Table 1:** Mesh convergence analysis.

Grid Density	Crack Depth	Amplitude ∆*B*	Relative Error
0.5 mm	1 mm	32.1 nT	-
0.2 mm	1 mm	33.8 nT	5.3%
0.1 mm	1 mm	34.1 nT	0.9%

**Table 2:** Grid meshing parameter table.

Area	Cell Size	Number of Elements
Air domain	2 mm	10,240
Aluminum alloy substrate	0.5 mm	8800
Weld seams and defects	0.1 mm	22,500

**Table 3:** The characteristic number of magnetic anomalies for cracks of different depths.

Crack Depth	Amplitude ∆*B*	Wave Width ∆*L*
0.4 mm	28.49 nT	2.85 mm
0.8 mm	30.41 nT	2.93 mm
1.2 mm	34.06 nT	2.95 mm
1.6 mm	35.44 nT	3.03 mm

**Table 4:** The characteristic number of magnetic anomalies for cracks of different lengths.

Crack Length	Amplitude ∆*B*	Wave Width ∆*L*
1 mm	32.61 nT	3.25 mm
2 mm	34.86 nT	4.18 mm
3 mm	35.72 nT	5.11 mm
4 mm	31.18 nT	6.24 mm

**Table 5:** The characteristic number of magnetic anomalies of holes of different diameters.

Pore	Amplitude ∆*B*	Pore Diameter *d*
pore1	5.2 nT	0.5 mm
pore2	9.8 nT	1.0 mm
pore3	14.5 nT	1.5 mm
pore4	19.3 nT	2.0 mm

**Table 6:** The chemical composition of 7A09 aluminum alloy (mass fraction %).

Elements	Cr	Cu	Mn	Ti	Zn	Mg	Si	Fe	Al
Mass Frac-tion %	0.15~0.30	1.2~2.0	≤0.10	≤0.10	5.1~6.1	2.0~3.0	≤0.50	0.45	≤87.4

**Table 7:** Amplitude and crack depth of each crack.

Crack	Amplitude ∆*B*	Crack Depth *h*
Crack 1	6.1 nT	1.2 mm
Crack 2	8.5 nT	1.6 mm
Crack 3	5.6 nT	0.8 mm
Crack 4	4.6 nT	0.4 mm

## Data Availability

Data are provided within the manuscript.

## Abbreviations

The following abbreviations are used in this manuscript:

NDT	Nondestructive testing
CT	Computed tomography
TOFD	Time-of-flight diffraction
CMS	Compact muon solenoid
